# Supplementary material for: Does menstrual hygiene management and water, sanitation, and hygiene predict reproductive tract infections among reproductive women in urban areas in Ethiopia?
Source: PLoS One. 2020 Aug 21;15(8):e0237696. doi: 10.1371/journal.pone.0237696 (PMC7444535; doi:10.1371/journal.pone.0237696)
Supplement: S1 File — (DOCX) [file pone.0237696.s001.docx]

## English version questionnaire

Questionnaires Code number ………Date of interview……….……time started…………….… Time finished………. …..

Full name of the data collector---------------------------------

1. **Socio-Demographic Factors Related To RTI**

| Sr. no | Questions | Response | Remark |
| --- | --- | --- | --- |
| 101 | Age | ___________year |  |
| 102 | Marital status | 1. Single 2. married 3. divorced 4. widowed |  |
| 103 | Religion | ___________________ |  |
| 104 | Educational status | __________ |  |
| 105 | Number of parity |  |  |

**Urban wealth index**

| 106  107  108  109  110  111  112 | What is the source of your drinking water? (more than one answer is possible )  1.houseline water Yes ... 1 No ... 0  2. pull and push/sway common water Yes ... 1 No ... 0  3. bono water Yes ... 1 No ... 0  4.protective pond water Yes ... 1 No ... 0  5.unprotective pond water Yes ... 1 No ... 0  6.stream water Yes ... 1 No ... 0  7. if others list…….. |  |
| --- | --- | --- |
| 113 | What type of toilet do you use?  1.Water Flush  2. Traditional toilet  3. Ventilated improved pit latrine  4. Open field  5. Others (list)……. |  |
| 114 | Who is the owner of your living house? 1. My own 2. rent house |  |
| 115 | Does your living house have dividing class? Yes ... 1 No ... 0 |  |
| 116 | Do you have a separated bedroom? Yes ... 1 No ... 0 |  |
| 117 | Do you have a separated kitchen? Yes ... 1 No ... 0 |  |
| 118  119  120  121  122 | From which material your house floor is made? (more than one answer is possible)  1. Natural ground Yes ... 1 No ... 0  2. Muck/smooth by cows faces Yes ... 1 No ... 0  3. Wood Yes ... 1 No ... 0  4. Cement Yes ... 1 No ... 0  5. if others list …….. |  |
| 123 | From which material your house roof is made? 1. Grass/ leaf 2. corrugated iron |  |
| 124  125  126  127  128 | From which material your house wall is made? (more than one answer is possible )  1. Wood but not have mod Yes ... 1 No ... 0  2. Wood with mod Yes ... 1 No ... 0  3. Wood and cement Yes ... 1 No ... 0  4. Blocket Yes ... 1 No ... 0  5. if others list…. |  |
| 129  130  131  132  133  134 | What is your energy source for food cooking? (more than one answer is possible )  1. Electric city system Yes ... 1 No ... 0  2. Gas /kerosene Yes ... 1 No ... 0  3. Wood /leaf Yes ... 1 No ... 0  4. Charcoal Yes ... 1 No ... 0  5. Animal feces Yes ... 1 No ... 0  6. If another list… |  |
| 135  136  137  138  139  140  141  142  143  144  145  146  147  148 | Among the following materials, which one do you own? (more than one answer is possible )  1. Radio Yes ... 1 No ... 0  2. Television Yes ... 1 No ... 0  3. House phone Yes ... 1 No ... 0  4. Fridge Yes ... 1 No ... 0  5. Chair Yes ... 1 No ... 0  6. Table Yes ... 1 No ... 0  7. Bed and mattress which made from cotton spring Yes ... 1 No ... 0  8. Mobile Yes ... 1 No ... 0  9. Cycle Yes ... 1 No ... 0  10. Motorcycle Yes ... 1 No ... 0  11. Horse’s cart Yes ... 1 No ... 0  12. Bajaj/car Yes ... 1 No ... 0  13.Bank book Yes ... 1 No ... 0  14. If another list…. |  |

1. **WaSH Related Factors**

| 201 | Presence of latrine/toilet for the household (with observation) | 1. no 2. yes |  |
| --- | --- | --- | --- |
| 202 | Is the latrine used utilized (with observation) | 1. no 1.yes |  |
| 203 | Cleanliness of latrine/toilet (with observation) | 1. Clean 2. not clean |  |
| 204 | Distance of latrine from the house | ________meter |  |
| 205 | Amount of water used in litter per capita per day | ______________ |  |
| 206 | Hand washing with soap before touching the genital area to clean | 1. no 2. yes |  |
| 207 | Presence of water near the toilet/latrine for hand washing | 1. no 2. yes |  |
| 208 | Water source | 1. house/yard connected tap water 2. public standpipe 3. well 4. spring 5. other __________ |  |

1. **History Of Co-Morbidities**

| 301 | History of abortion | 1. no 2. yes |  |
| --- | --- | --- | --- |
| 302 | itching, ulcer around the vagina, yellowish or greenish or white with burning sensation vaginal discharge, pain during urination in the past one year before three months from now | 1. no 2. yes |  |

1. **Behavioral Factors**

| 401 | Multiple sexual partner | 1. no 1. Yes |  |
| --- | --- | --- | --- |
| 402 | Sexual intercourse during menstruation | 1. no 1. Yes |  |
| 403 | Type of contraceptive used | 1. IUCD 2. condom 3. dipo 4. injectable 5. other______ |  |

1. **MHM Related factors**

| 501 | Did you used blood absorbent material during menstruation | 1. no 2. yes | ≠507 |
| --- | --- | --- | --- |
| 502 | Nature of material used during menstruation | 1. Sanitary pad/ napkins(hygienic) 2. Old cloth(unhygienic) 3. other(list)___________ |  |
| 503 | Where do you put your menstruation pad/cloth? | 1. I reuse it 2. I dispose of it | ≠505 |
| 504 | Frequency of the day for changing of absorbent material during menstruation | _________________ |  |
| 505 | frequency of changing of absorbent material per day during menstruation | __________________________ |  |
| 506 | Did you take all body bath every day during menstruation | 1. no 1. yes |  |
| 507 | When you take all body bath during menstruation days | 1. alternative day 2. every day 3. other_____ |  |
| 508 | Did you wash the genital area every day during menstruation | 1. no 2. yes | ≠ 601 |
| 509 | How many times did you wash genital area per day during menstruation | _______________________ |  |
| 510 | What do you use to wash the genital area?  (do not read the choice) | 1. water only 2. water and soap 3. other_________ |  |

1. **Symptoms Observed In The Past Three Months Period**

|  | Anyone of the following symptoms in the past three months | |  |
| --- | --- | --- | --- |
| 601 | Yellowish or greenish or white type with burning sensation Vaginal discharge | 1. no 2. yes |  |
| 602 | Burning sensation during urination | 1. no 2. yes |  |
| 603 | Itching/irritation around the genitalia | 1. no 2. yes |  |
| 604 | lower back Pain | 1. no 2. yes |  |
| 605 | lower abdomen Pain | 1. no 2. yes |  |
| 606 | Genital ulcers/ lesion | 1. no 2. yes |  |
| 607 | Reproductive tract infection | 1. no 2. yes |  |

**Thank you for your valuable information**
