# Supplementary material for: Does menstrual hygiene management and water, sanitation, and hygiene predict reproductive tract infections among reproductive women in urban areas in Ethiopia?
Source: PLoS One. 2020 Aug 21;15(8):e0237696. doi: 10.1371/journal.pone.0237696 (PMC7444535; doi:10.1371/journal.pone.0237696)
Supplement: S2 File — (DOCX) [file pone.0237696.s002.docx]

## Amharic version questionnaire

የመጠይቁ ኮድ--------------------ቃለ መጠይቁ የተካሄደበት ቀን----------------------ቃለ መጠይቁ የተጀመረበት ጊዜ---------------ቃለ መጠይቁ የተጨረሰበት ጊዜ---------

የመረጃ ሰብሳቢው ሙሉ ስም ---------------------------------------------

**ማህበራዊ ኢኮኖሚያዊ እና ስነህዝባዊ ጉዳች ጋር የተያያዙ ጥያቄወች**

| ተ.ቁ | ጥያቄ | መልስ | Remark |
| --- | --- | --- | --- |
| 101 | ዕድሜ | ________ዓመት |  |
| 102 | የጋብቻ ሁኔታ | 1. ያላገባች 2. ያገባች 3. የፈታች 4. ባሏየሞተባት |  |
| 103 | ሀይማኖት | __________________ |  |
| 104 | የትምህርት ደረጃ | ___________________ |  |
| 105 | ስንት ጊዜ ወልደሻል | __________________ |  |

**የከተማ ሐብት ሁኔታ ጠቋሚ መረጃ**

| 106  107  108  109  110  111  112 | የምትጠቀሙት ዉሃ መገኛው ከየት ነዉ? (ከአንድ በላይ መመለስ ይቻላል)  1. በቤት ዉስጥ ያለ የመስመር ዉሃ አወ …..1 የለም …..0  2. በእጅ የሚነቀነቅ የጋራዉሃ አወ …..1 የለም….. 0  3. ቦኖ ዉሃ አወ …..1 የለም…..0  4. የተከለለ የጉድጋድ ዉሃ አወ …..1 የለም….. 0  5 ያልተከለለ የጉድጋድ ዉሃ አወ …..1 የለም….. 0  6. የምንጭ /የወራጅ/ ኩሬ የግድብ ዉሃ አወ …..1 የለም….. 0  7. ሌላ ካለ ይጥቀሱ ________ |  |
| --- | --- | --- |
| 113 | የምትጠቀሙበት መጸዳጃ ቤት ምን አይነት ነዉ?  1. በዉሃ የሚሰራ ሽንት ቤት  2. ባህላዊ ሽንት ቤት  3. የአየር ማስወጫ ቱቦ ያለዉ ሽንት ቤት  4. ሜዳ ላይ  5. ሌላ ካለ ይጥቀሱ ------- |  |
| 114 | የምትኖሩበት ቤት ባለቤቱ ማን ነዉ? 1. የግልዎ 2. ተከራይተው |  |
| 115 | መኖሪያ ቤታችሁ የተለያ የአገልግሎት እንድሰጥ የተከፋፈለ ነውን? አወ…..1 የለም…..0 |  |
| 116 | የተለየ የምኝታ ክፍል ለብቻ አለወይ? አወ….1 የለም….0 |  |
| 117 | የተለየ የምግብ ማብሰያ /ኩሽና) ክፍል አለ ወይ? አወ…..1 የለም…..0 |  |
| 118  119  120  121  122 | የቤቱ ወለል ከምንድን ነዉ የተሰራዉ? (ከአንድ በላይ መመለስ ይቻላል)  1. የተፈጥሮ መሬት/ወለሉ ምንምነገር የሌለዉ አወ …..1 የለም….. 0  2. በከብቶች እበት የተሰራ /የተለቀለቀ ወለል አወ …..1 የለም….. 0  3. በእንጨት የተሰራ ወለል አወ …..1 የለም….. 0  4. በሲሚንቶ የተሰራ ወለል አወ …..1 የለም….. 0  5. ሌላ ካለይ ጥቀሱ….. |  |
| 123 | የቤቱ ጣራ ከምድን ነዉ የተሰራዉ? 1. ከሳር/ከቅጠል 2. ከቆርቆሮ |  |
| 124  125  126  127  128 | የቤቱግድግዳ ከምንድን ነዉ የተሰራዉ? (ከአንድ በላይ መመለስ ይቻላል)  1. ከእንጨት ሁኖ ጭቃ የሌለዉ አወ …..1 የለም….. 0  2. ከእንጨት እና ከጭቃ አወ …..1 የለም….. 0  3. ከእንጨት እና ከሲሚነቶ አወ …..1 የለም …..0  4. ከብሎኬት የተሰራ አወ…..1 የለም….. 0  5. ሌላ ካለ ይጥቀሱ…… |  |
| 129  130  131  132  133  134 | ለምግብ ማብሰያነት የምትጠቀሙበት የሀይል ምንጭ ምንድን ነዉ? (ከአንድ በላይ መመለስ ይቻላል)  1. ኤሌክትሪክ አወ… 1 የለም….. 0  2. ነጭ ጋዝ አወ… 1 የለም….. 0  3. እንጨት /ቅጠል አወ… 1 የለም….. 0  4. ከሰል አወ… 1 የለም….. 0  5. ኩበት/በጠጥ አወ… 1 የለም….. 0  6. ሌላ ካለ ይጥቀሱ…… |  |
|  | ክዚህ በታች ከተዘረዘሩት ንብረቶች በቤታችሁ ዉስጥ ያላችሁ የቱ ነዉ? (ከአንድ በላይ መመለስይቻላል) |  |
| 135  136  137  138  139  140  141  142  143  144  145  146  147  148 | 1. ሬደዮ አወ…….1 የለም……..0  2.ቴሌቪዥን አወ……1 የለም……..0  3. የቤት ስልክ አወ…….1 የለም…….0  4. ፍሪጅ አወ……1 የለም..……0  5. ወንበር አወ…….1 የለም…….0  6. ጠረንጴዛ አወ…….1 የለም…….0  7. የጥጥ/የእስቦንጅ / አስፕሪነግ ፍራሽ ያለዉ አልጋ አወ……1 የለም….0  8. ሞባይል ስልክ አወ………1 የለም…….0  9. ሳይክል አወ………1 የለም..…..0  10. ሞተር ሳይክል አወ………1 የለም…….0  11. የፈረስ ጋሪ አወ………1 የለም……0  12. ባጃጅ/መኪና አወ………1 የለም……0  13. የባንክ ቡክ አወ………1 የለም……0  14. ሌላ ካለይ ጥቀሱ…… |  |

**ከዉሃ እና መጸዳጃ ቤት ጋር የተያያዙ ጥያቄዎች**

| 201 | ቤቱ መጸዳጃ አለው ወይ **(በምልከታ)** | 1. የለውም 1. አዎ |  |
| --- | --- | --- | --- |
| 202 | መጸዳጃ ቤቱን ይጠቀሙበታል ወይ **(በምልከታ)** | 1. የለም 1. አዎ |  |
| 203 | የመጸዳጃ ቤቱ የጽዳት ሁኔታ **(በምልከታ)** | 1. ንጹህ 1. ንጹህ ያልሆነ |  |
| 204 | መጸዳጃ ቤቱ ያለው እርቀት በሜትር | ___________________ |  |
| 205 | በቀን አንድ የቤተሰብ አባል የሚጠቀመው የውሃ መጠን በሊትር | ________________ |  |
| 206 | ብልትን ከማጽዳትሽ በፊት እጅሽን በውሃና በሳሙና ትታጠቢያለሽ ወይ | 1. የለም 1. አዎ |  |
| 207 | ከመጸጸዳጃ ቤቱ አካባቢ ለእጅ መታጠቢያ ዉሃ አለ ወይ **(በምልከታ)** | 1. የለም 1. አዎ |  |
| 208 | የውሃ ምንጫችሁ ከየት ነው | 1. ቤት/ግቢ ውስጥ ያለ ቧንቧ 2. ቦኖ 3. ጉድጓድ ውሃ 4. የምንጭ ውሃ 5. ሌላ ካለይገለጽ---------- |  |

**ከአሁን በፊት ከነበሩ ጉዳዮች ጋር የተያያዙ ጥያቄወች**

| 301 | የጽንስ ማቋረጥ አድርገሽ ታውቂያለሽ ወይ | 1. የለም 2. አዎ |  |
| --- | --- | --- | --- |
| 302 | ከሶስት ወር በፊት ባለው ባለፈው አንድ ዓመት ውስጥ በብልት አካባቢ የማሳከክ ሁኔታና ቁስለት፣በመሽናት ጊዜ የማቃጠል ስሜት፣ በብልት የሚወጣ ቢጫማ ወይም አረንጓዴማ ወይም ነጭ ፈሳሽ ከማቃጠል ስሜት ጋር የተያያዘ ፈሳሽ ነገር ፣ ነበር ወይ | 1. የለም 2. አዎ |  |

**ከባህርይ ጋር የተያያዙ ጥያቄዎች**

| 401 | ከአንድ በላይ የግብረ ስጋ ግንኙነት ጓደኛ አለሽ ወይ | 1. የለም 1. አዎ |  |
| --- | --- | --- | --- |
| 402 | በወር አበባ ወቅት የግብረ ስጋ ግንኙነት ታደርጊያለሽ ወይ | 1. የለም 1. አዎ |  |
| 403 | የምትጠቀሚው የወሊድ መከላከያ አይነት | 1. በማህጸን የሚቀበር 2. ኮንዶም 3. በክንድ የሚቀበር 4. መርፌ 5. ሌላ ካለይገለጽ   -------------------------- |  |

**በወር አበባ ጊዜ የሚደረግ ንጽህና አጠባበቅ ጋር የተያያዙ ጥያቄወች**

| 501 | በወር አበባ ወቅት ፈሳሽ ለመምጠጥ የንጽህና መጠበቂያ ትጠቀሚያለሽ ወይ | 1. የለም 2. አዎ | ≠ 507 |
| --- | --- | --- | --- |
| 502 | በወር አበባ ወቅት የምትጠቀሚው የንጽህና መጠበቂያ ስሪቱ ምንድን ነው | 1. ሞዴስ 2. አሮጌ ጨርቅ 3. ሌላ ካለይገለጽ-------------------------- |  |
| 503 | በወር አበባ ወቅት የተጠቀምሽበትን የንጽህና መጠበቂያ የት ታስቀምጫለሽ | 1. እንደገና እጠቀምበታለሁ 2. አስወግደዋለሁ/እጥለዋለሁ | ≠505 |
| 504 | የንጽህና መጠበቂያውን በየስንት ቀን ትቀይሪያለሽ | ___________________ |  |
| 505 | በወር አበባ ወቅት በቀን ስንት ጊዜ የንጽህና መጠበቂያውን ትቀይሪያለሽ | _____________________ |  |
| 506 | በወር አበባ ወቅት መላው ሰውነትሽን በየስንት ቀኑ ትታጠቢያለሽ | 1. የለም 2. አዎ |  |
| 507 | በየስንት ቀኑ ትታጠቢያለሽ | 1. በአንድ ቀን ልዩነት 2. በየቀኑ 3. ሌላ------------------- |  |
| 508 | በወር አበባ ወቅት ብልትሽን በየቀኑ ትታጠቢያለሽ ወይ | 1. የለም 2. አዎ | ≠ 601 |
| 509 | በወር አበባ ወቅት ብልትሽን በቀን ስንት ጊዜ ትታጠቢያለሽ | ___________________________ |  |
| 510 | ብልት ለመታጠብ የምትጠቀሚው ምንድን ነው **(ምርጫው አይነበብም)** | 1. ውሃ ብቻ 2. ውሃና ሳሙና 3. ሌላ ካለይገለጽ-------------------------- |  |

**ከበሽታ ምልክት ጋር የተያያዙ ጥያቄዎች**

|  | ከታች የተዘረዘሩት ምልክቶች ባለፈው 3 ወር ተከስቶብሻል | |  |
| --- | --- | --- | --- |
| 601 | በብልት የሚወጣ ቢጫማ ወይም አረንጓዴ ወይም ነጭ ፍሳሽ ነገር ከማቃጠል ስሜት ጋር የተቀላቀለ አይነት ፈሳሽ ነገር | 1. የለም 2. አዎ |  |
| 602 | በሽንት ወቅት የማቃጠል ስሜት | 1. የለም 2. አዎ |  |
| 603 | ብልት አካባቢ የማሳከክ ሁኔታ | 1. የለም 2. አዎ |  |
| 604 | የኋላ ታች ወገብ ህመም | 1. የለም 2. አዎ |  |
| 605 | ወደታች ሆድ አካባቢ ህመም | 1. የለም 2. አዎ |  |
| 606 | ብልት አካባቢ ቁስለት | 1. የለም 2. አዎ |  |
| 607 | በብልት አካባቢ ማመርቀዝ በሽታ | 1. የለም 2. አዎ |  |

ስለተሳትፎዎት አመሰግናለሁ!
